# Do Objective or Subjective Neighborhood Indicators Protect Against Adversity on Mental Health and Well-Being?

**DOI:** 10.1093/geroni/igab046.1514

**Published:** 2021-12-17

**Authors:** Omar Staben, Frank Infurna, Kevin Grimm, Suniya Luthar

**Affiliations:** Arizona State University, Tempe, Arizona, United States

## Abstract

The neighborhood context through which individuals interact is shown to be associated with mental and physical health across adulthood. Much less is known regarding potential underlying reasons why, such as protecting against the deleterious effects of stress. This study explores whether objective and subjective neighborhood factors are associated with maintenance of mental health and well-being in the context of monthly adversity. We use longitudinal data from a sample of midlife (N =362) who completed monthly questionnaires for two years. Results show that experiencing a monthly adversity was associated with poorer mental health and well-being. Living in a neighborhood with more disorder was associated with stronger declines in mental health and well-being when a monthly adversity was reported. Our discussion focuses on why the neighborhood context is relevant for middle-aged adults and the various ways through which neighborhood context has the potential to shape the course of development in adulthood.

